# Sugar-Sweetened Beverages Contribute Significantly to College Students’ Daily Caloric Intake in Jordan: Soft Drinks Are Not the Major Contributor

**DOI:** 10.3390/nu11051058

**Published:** 2019-05-11

**Authors:** Hiba Bawadi, Toqa Khataybeh, Bayan Obeidat, Abdelhamid Kerkadi, Reema Tayyem, Angela D. Banks, Hadil Subih

**Affiliations:** 1 Department of Nutrition, College of Health Sciences, QU-Health, Qatar University, Doha P.O.Box 2713, Qatar; abdel.hamid@qu.edu.qa; 2 Department of Nutrition and Food Technology, Jordan University of Science and Technology, Irbid 22110, Jordan; t.khataybeh@yahoo.com (T.K.); obeidatb@just.edu.jo (B.O.); hssubih@just.edu.jo (H.S.); 3 Department of Nutrition and Food Technology, Faculty of Agriculture, University of Jordan, Amman 11942, Jordan; R.Tayyem@ju.edu.jo; 4 USF School of Nursing and Health Professions, San Francisco, CA 94117-1080, USA; adbanks@usfca.edu

**Keywords:** sugar sweetened beverages, Jordan, body weight

## Abstract

Sugar sweetened beverages (SSBs) are caloric-dense and associated with poor diet quality which may result in weight gain and obesity. Obesity is an independent risk factor for several chronic diseases. This study aimed to (1) assess the consumption level of SSBs among college students in Jordan and (2) examine the relationship of consumption level to body weight. The current study is a cross-sectional study conducted on 967 college students (55.3% males and 44.7% females). Consumption of SSBs was assessed using validated questionnaires. SSBs were classified into four major categories (hot drinks, fruit drinks, energy drinks, and soft drinks). Anthropometric measurements of the participants including body weight, height, and waist circumferences were recorded. Male students consumed more calories from SSBs compared to female students (*p* = 0.016). The mean contribution of SSBs to daily energy intake among college students was 480 kcal with the highest contribution from sugar sweetened hot drinks and fruit drinks. A significant positive relationship was found in BMI (*p* = 0.006) and waist circumference (*p* = 0.030) for participants consuming calories from SSBs. In conclusion, beverages with added sugar contribute substantially to the daily energy intake of college students in Jordan.

## 1. Introduction

High intake of added sugar is a major characteristic of poor diet and an unhealthy life style that may be associated with increased prevalence of obesity and overweight [1]. The World Health Organization (WHO) strongly recommends reducing the intake of added sugar to control the obesity epidemic [2]. Sugar sweetened beverages (SSBs) are considered major sources of added sugar in the diet [3]. The Center for Disease Control and Prevention defines SSBs as drinks with added sugar in form of table sugar or high fructose corn syrup [4]. SSBs include—but are not limited to—soft drinks (sodas), sports drinks, fruit drinks, tea and coffee drinks, energy drinks, sweetened milk or milk alternatives [4]. Several studies have illustrated the association between SSB intake and non-communicable diseases [5], such as type 2 diabetes [6,7] and cardiometabolic disease [8,9]. The harmful health effects from SSBs may be explained by the high content of calories which may increase the risk of weight gain and obesity [10] and by the substitution of nutritive beverages such as 100% fruit juice and milk with empty calories from SSBs [11]. Studies indicate that individuals consuming high amounts of SSBs usually have low intakes of Vitamin C, Vitamin A, riboflavin, magnesium, calcium, and fiber [12].

In recent years, SSBs consumption has dramatically increased worldwide particularly among young adults [13,14]. The high consumption of SSBs is associated with weight gain and obesity among children and adults [15]. The proposed mechanism to explain the association between SSBs consumption and weight gain is that consumption of liquid calories from SSBs has a weak satiety effect compared to consumption of calories from solid foods and may result in excess intake of calories [16,17]. In addition, the results of the PREMIER trial found that a reduction of SSBs intake among adults was significantly associated with weight loss [18]. A reduction in intake of one serving/d of SSB was associated with a 0.5 kg weight loss at six months and a 0.7 kg weight loss at 18 months [18].

As overweight and obesity are a major health problem among Jordanian college students [19] and the data concerning the consumption of SSBs in college students in Jordan is very limited, it is important to examine the association between the consumption of SSBs and body weight. Thus, the aim of this study is to assess the consumption pattern of SSBs among college students and its impact on their body weight.

## 2. Methods

### 2.1. Study Participants

A convenient sample of 1000 students aged between 18 and 25 years was obtained from a major university in Jordan. The main assumption made for convenience sampling was that our population (college students in a major governmental university) is homogenous [20]. Limitations associated with convenience sampling are discussed further below. Inclusion criteria consisted of healthy Jordanian students over the age of 18. Students were invited to participate and the objectives of the study were explained to each participant. Students were asked to sign an informed consent form. The Institutional Review Board at Jordan University of Science and Technology reviewed and approved the methods and tools of this study. A total of 967 of 1000 students completed the questionnaires, for a response rate of 96.7%.

### 2.2. Questionnaires

Three self-administered questionnaires were used in this study: (1) demographic and health status questionnaire, (2) physical activity questionnaire, and (3) SSBs consumption frequency questionnaire. Sociodemographic and health status questionnaires were used to collect information on age, gender, education level, household income, health status, and smoking status.

### 2.3. Assessment of Physical Activity

Participants’ physical activity was assessed using the short form of the International Physical Activity Questionnaire (IPAQ). Short IPAQ is a set of 7 questions related to vigorous activity, moderate activity, walking, and sitting during the last 7 days. IPAQ data were analyzed using a standard scoring protocol. The metabolic equivalent (MET) was also obtained. Average MET values were derived for each type of activity during the IPAQ reliability study [21]. MET scores were provided for each kind of physical activity. Total MET-min per week was obtained as MET level for activity multiplied by duration of activity (minutes)/day multiplied by the number of days the activity is practiced per week. Three levels of physical activity were categorized based on MET-min per week as inactive, minimally active, and Health Enhancing Physical Activity (HEPA) active [21]. A low physical activity level was assigned when individuals did not meet the criteria set for moderate or high physical activity. Moderate physical activity was assigned if participants (a) practiced at least 20 min of vigorous-intensity activity for 3 or more days per week, (b) practiced 30 min of walking/moderate-intensity physical activity for 5 or more days per week, or (c) achieved at least 600 MET-minutes/week from a combination of walking at a moderate or vigorous intensity. A high physical activity level was assigned if participants (a) achieved a minimum of 1500 MET-minutes/week from vigorous-intensity activity at least 3 days per week or (b) achieved a minimum of 3000 MET-minutes/week from a combination of walking, moderate-intensity, or vigorous-intensity 7 days per week.

### 2.4. Assessment of SSBs Consumption

A sugar-sweetened beverage in this study is defined as a drink with sugar added during processing and preparation. Screening and listing of beverages sold in campus cafeterias, coffee shops, kiosks, and grocery stores were conducted by the researchers. The beverages included sweetened American coffee, sweetened latte, cappuccino, mochaccino, macchiato, instant coffee 3 in 1/2 in 1, sweetened instant coffee with milk, hot chocolate, frozen drinks (slush), energy drinks, soft (carbonated) beverages, fruit drinks, flavored milk, sweetened tea, and sweetened brewed coffee. Data regarding the amount of added sugar in home prepared SSBs such as teas and coffees were also collected. For each home prepared SSB, participants were asked to choose one of three levels of sweetness; (1) 1 teaspoon of added sugar/240 mL of drink; (2) 2 teaspoons of added sugar/240 mL of drink; and (3)3 teaspoons of added sugar/240 mL of drink. For each type of previously screened beverage, choices of 9 frequencies of consumption were provided. The range was no consumption to 6 times/day. Participants were asked to choose their level of consumption from the listed items. Samples of commonly sold SSBs were provided to participants to assist them in estimating their portion size. Participants were allowed to add SSBs consumed, but not included in the list. In addition, they were also asked to provide commercial brand names of these items. The SSBs consumption frequency questionnaire was assessed for reliability on a pilot of 50 students (not included in the study sample). The questionnaire was administered twice within a 2-week period. Pearson’s coefficient for the test-retest was 0.95 (*p* = 0.001).

The validity of the SSBs consumption frequency questionnaire was tested against a 24 h recall collected from all participants. The average daily consumption of SSBs from the FFQ was validated against that from a 24 h recall. Cronbach’s alpha = 0.78. Participants’ 24 h food recall was later used to estimate total daily caloric consumption (to be adjusted for in the statistical analysis).

### 2.5. Anthropometric Measurements

Weight, height, and waist circumference were measured by trained personnel. Height was measured using measuring tape (Seca 201, Hamburg, Germany). Students were asked to remove any hair ornaments and align the head in a Frankfort horizontal plane. Then they were asked to take off their shoes while standing with heels together and toes apart, ensuring that the back of the head, shoulder blades, buttocks, and heels were in contact with the backboard if possible. Body weight of the participant was measured while dressed in light clothes and without shoes using the Omron body fat monitor (BF400 Tokyo, Japan) [22]. Waist circumference was measured using circumference measuring tape (Seca 200, Hamburg, Germany). The cut-off points for waist circumference were high if >102 cm in males, and >88 cm in females [23]. Body mass index (BMI) was calculated and body weight categories were established based on WHO guidelines.

### 2.6. Statistical Analysis

Weekly and monthly reported consumption of SSBs was transformed into daily consumption. For frequency consumption with range, the midpoint value was used. Descriptive analyses were conducted where means ± standard deviation were used to describe continuous variables and frequency (percentage) were used to describe categorical variables. A chi-square test was conducted to examine the distribution of student’s socio-demographic characteristics at different SSB consumption levels. Participants were categorized into tertiles of calorie consumption from SSBs. Differences in participants’ BMI and WC across different tertiles were examined using univariate analysis of variance. These were adjusted for several covariates including gender (males, females); total daily calories (continuous variable); physical activity (categorical: low, moderate, and high physical activity); and smoking (smoker, non-smoker). Mean analysis of BMI and WC across the different SSBs tertile groups was conducted to observe a possible dose–response relationship.

## 3. Results

This study investigated the consumption of SSBs among Jordanian college students and the effect of their overconsumption on body weight. Table 1 shows the socio-demographic characteristics of the study sample. The majority of students (82.8%) were living with their families. Most of the students were coming from families with incomes between 200–500 JD. About one third of participants were smokers and about 66% reported moderate physical activity. Regarding body weight, about 28% of the students were either overweight or obese.

Table 2 shows students’ caloric consumption from SSBs. The most frequently consumed SSB category was hot drinks with an average of four servings/day contributing to 180 calories/day. Fruit drinks were less frequently consumed but contributed significantly to equivalent daily calories as hot drinks. The mean total caloric intake from SSBs was 481 kcal per day. Students’ consumption of SSBs according to their sociodemographic characteristics is presented in Table 3. Male students consumed higher calories from SSBs than females (532.4 vs. 416.6 kcal/day respectively; *p* = 0.016). Smoking status of the students was also associated with the consumption of SSBs (*p* < 0.05). Smokers consume higher amounts of SSBs compared to nonsmokers (585.3 vs 450.0 kcal/day respectively). No differences were observed in mean caloric consumption from SSBs between students who lived with their families and those who lived in student dormitories (*p* < 0.05).

Table 4 shows the association between caloric consumption from SSBs on body weight parameters. Our results indicate that BMI and WC were positively associated with caloric consumption of SSBs in a dose-response relationship. Students’ BMI and WC increased across tertile 2 and tertile 3 on caloric consumption from SSBs.

## 4. Discussion

This study investigated the consumption of SSBs among Jordanian college students and the effect of overconsumption on body weight. The study is considered the first one among college students in Jordan.

Results of the present study indicate that 60% of students reported drinking SSBs daily. The highest rate of SSB consumption was for hot drinks (89.1%) followed by fruit drinks (45.3%), and soda (45.2%). Only 1.2% of students reported drinking a can, bottle, or a glass of energy drink ≥1 time/day. The prevalence of a student consuming some form of SSB was lower than that reported in USA [24,25], Canada [26], Belgium [27], and Bangladesh [28].

In the overall sample of Jordanian students, the average consumption of SSB was 1.53 servings per day. Results of our study were higher than those reported by other studies [29,30]. Results of the global survey data on individual SSB consumption in adults over age 20 showed an average of 0.58 servings per day [30]. Compared to the United States, the adjusted prevalence of SSB consumption by young adults was 0.73 in 2007–2008 [30].

In the study population, the mean caloric intake from SSB was 480.6 kcal/d of which 179.8 kcal/d, 180.7 kcal/d, and 133.6 kcal/d were from hot drinks, fruit drinks, and soda, respectively. Our results are higher than that reported in the USA [29,31] and Mexico [32,33]. Bipasha et al. (2017) in Bangladesh found similar results [28]. Discrepancies between studies could be attributed to a difference in study population (adults, college students, adolescents, and children) or types of SSB included in the definition (Sweetened soft drinks, milk, juice, soda, fruit drinks, spirits).

Globally, the most consumed SSBs are soft and energy drinks. We found that the intake of soft and energy drinks combined correspond to an estimated calorie intake of 139 kcal/d. This amount is lower than that reported in the USA [27] and in Belgium [34]. We noted that energy drinks were not highly consumed in Jordan. The estimated calorie intake from SSB represented 22% of the total energy requirement for this age group. Studies in the United States, France, and Italy [31,35] have shown different results to ours while in Belgium, Spain, the Netherlands, and Mexico [33,35] the contribution of SSB intake to the total calorie intake was similar to ours.

In this study males consumed greater amounts of SSBs than females (*p* < 0.05). Similar findings were reported previously. A research using a food frequency questionnaire to assess the SSBs intake of 265 undergraduates at the University of Arkansas (2006) showed that males were more likely than females to report daily intake of SSBs [27]. Similar findings were reported by a study conducted by Gomez et al. (2009) [36] in which males (*n* = 768) consumed higher quantities of SSBs than females (*n* = 755), with an average caloric intake of 157.8 ± 164.5 and 71.6 [21] ± 113.5, respectively. Based on the results of the aforementioned studies, it is possible that males consumed higher amounts of SSBs than females because they are less concerned with their body image compared to females [11,37]. Other studies indicated that the consumption of sugar sweetened beverages is higher in males compared to females as men consume less water than females as reported by Xi and colleagues [38]. Moreover, the overall food consumption among males is higher than among females and hence it is expected that males’ total consumption of SSBs is higher [37].

Daily cigarette smoking was reported by 22.6% of students. Several studies have investigated the associations between SSB intake and some unhealthy behaviors such as smoking and physical inactivity [39,40,41,42]. We observed statistically significant differences in caloric intake from SSB between smokers and non-smokers (*p* = 0.009). Similarly, Lee et al. also reported that the frequency of SSB consumption was strongly associated with smoking behaviors [40]. Their researchers found that non-daily consumers of SSB had lower odds of being smokers than those with daily consumption (OR: 0.46, 95% CI = 0.31–0.68). Another study revealed that the odds of being in the highest tertile for added sugar intake were associated with being a smoker (OR: 1.96, 95% IC = 1.66–2.30) [41]. Additionally, a positive association between soda intake and cigarette smoking has been previously reported [43,44]. Different mechanisms have been suggested to explain the association between SSB and cigarettes. Previous studies have shown that smokers may have decreased sensitivity to sucrose, suggesting that smokers may be less likely to perceive food as being sweet and thus eat more [45,46]. Taste perception among smokers may be altered because of poor oral hygiene [47] or damage to the oral peripheral tissues [48].

In the current study, students who live with their parents did not seem to consume different amounts of calories from SSBs compared to students who live in dorms. Previous studies showed different results [49]; students who live with their families seem to consume less SSBs than those who live on campus. These conflicting results may be explained by the differences in the type of SSBs consumed. As discussed earlier, the most frequently consumed SSBs in Jordan were sugar sweetened hot drinks unlike the soft drinks in Western studies. Culturally, tea and coffee are prepared during family times which may explain why students living with their families consume greater amounts of SSBs.

In the current study, BMI and calories from SSB were significantly related (*p* = 0.006). The relationship between SSBs consumption and overweight is supported by numerous studies [50,51,52,53,54,55]. In a randomized controlled interventional study conducted on 810 participants, reduction in the consumption of SSBs resulted on weight loss at 6 and 18 months post intervention [18]. Raben et al. (2002) conducted a 10 week weight loss trial. The results revealed that a study group that consumed SSBs had an increased fat accumulation (1.3 ± 0.5 kg) and a higher body weight (1.6 ± 0.4 kg) [56]. Bermudez and colleagues [12] investigated the association between SSB consumption and abdominal obesity for 947 participants using the 1999–2000 NHANES data. The researchers observed that high SSB consumers had a substantially larger waist circumference and BMI compared to low SSB consumers [12]. Findings from a longitudinal study show that for every additional fluid ounce of SSBs from baseline there was a simultaneous increase in BMI [53].

Many explanations were proposed to clarify the relationship between SSB consumption and BMI. Proposed explanations include (1) the higher caloric intake from SSBs that may result in positive energy balance and increased adiposity [57], (2) the decrease in satiety resulting from the fast digestion of liquid calories [16,17], and (3) the higher lipogenic effect of fructose and its impact on insulin and leptin secretion [58].

Findings of this study may be limited due to the self-reporting of data by the participants, which is subject to recall bias and portion size estimate errors. Another limitation of this study is the use of a single 24 h recall to validate the FFQ of SSBs consumption; the use of food records could have been more accurate. The generalizability of the study’s findings to the general adult population in Jordan may also be limited because adults in this study were young adults attending college. Limitations associated with convenience sampling—besides limited generalizability—include possible risk of bias due to self-selection [20].

In conclusion, SSBs contribute about 500 kcal per day with sugar sweetened hot drinks being the most frequently consumed. Greater attention must be given to sugar content in home prepared hot drinks.

## Figures and Tables

**Table 1:** Sample socio-demographic and anthropometric characteristics (*n* = 967).

	*n* (%)
**Gender**	
Male	535 (55.3)
Female	432 (44.7)
**Student’s residency**	
With parents	801 (82.8)
Students’ dorms	166 (17.2)
Family Income (JD) *	
<200	59 (6.1)
200–500	427 (44.2)
500–1000	322 (33.3)
>1000	159 (16.4)
**Smoking Status**	
Smoker	219 (22.6)
Nonsmoker	748 (77.4)
**Physical Activity ^†^**	
Low Physical Activity	59 (6.1)
Moderate Physical Activity	637 (65.9)
High Physical Activity	271 (28.0)
Body Mass Index (BMI) ^‡^	
Underweight	95 (9.8)
Normal	606 (62.7)
Overweight	201 (20.8)
Obese	65 (6.7)
**Waist Circumference (WC) ^§^**	
Normal	909 (94.4)
At risk	58 (5.5)

* Jordanian dinar (JD) = $1.4; **^†^** Physical activity levels were established based on IPAQ standard protocol [15]; ^‡^ Underweight (less than 18.5 kg/m^2^), normal weight (18.6–24.9 kg /m^2^), overweight (25–29.9 kg/m^2^), obese (BMI ≥ 30); ^§^ High waist circumference when >102 cm in males, and >88 cm in females.

**Table 2:** Calories and servings consumed per day from SSBs among participants.

	Calories ^†^ Mean ± SD	Servings Mean ± SD
**Hot drinks ^‡^**	179.8 ± 159.1	4.0 ± 3.0
**Fruit drinks ^¶^**	180.7 ± 193.9	1.0 ± 1.1
**Energy drinks**	4.9 ± 18.8	0.04 ± 0.16
**Soft drinks**	133.6 ± 145.6	1.1 ± 1.2
**Total calories**	480.6 ± 338.89	

**^†^** Calories for each item in each category were reported and summed to get the mean caloric contribution. **^‡^** Hot drinks include American coffee, milk coffee, cappuccino, mochaccino, macchiato, instant coffee 3-in-1, milk with Nescafe, hot chocolate, milk, sweetened tea and sweetened brewed coffee; ^¶^ Fruit drinks include fruit punch; fruit-flavored drinks and processed fruit juices with added sugar.

**Table 3:** Calories from SSBs in relation to students’ characteristics.

Variable	Mean ± SD	*p*-Value
**Gender**		0.016
Male	532.4 ± 343.9	
Female	416.6 ± 321.6	
**Students residency**		0.6
With parents	483.2± 345.6	
Students’ dorms	468.3± 305.0	
Family income (JD) *		0.617
<200	453.6 ± 360.6	
200–500	470.1 ± 327.4	
500–1000	474.5 ± 321.9	
>1000	551.5 ± 389.3	
**Smoking status**		0.009
Smoker	535.3 ± 363.3	
Nonsmoker	450.0 ± 325.3	
**Physical activity**		0.784
Low Physical Activity (inactive)	429.3 ± 333.0	
Moderate Physical Activity (minimally active)	477.4 ± 340.2	
High Physical Activity (HEPA)	± 337.0	

* JD (Jordanian Dinar) = 1.41 USD.

**Table 4:** Association between calorie consumption from SSBs and body weight parameters ^†^.

Weight Parameters	Tertile1 ^‡^	Tertile2 ^¶^	Tertile3 ^§^	*p*-Value
	Mean ± SD	Mean ± SD	Mean ± SD	
**BMI**	22.4 ± 3.7 ^a^	23.2 ± 4.0 ^b^	24.0 ± 4.5 ^c^	0.006
**Waist circumference**	75.1 ± 12.4 ^a^	78.3 ± 13.5 ^b^	81.3 ± 15.1 ^c^	0.030

^†^ Controlled for physical activity, gender, total calories and smoking; Different superscripts donate the statistical differences contained in the row; ^‡^ calories of SSB is less than 285.6; ^¶^ calories of SSB is between 285.7 and 543; ^§^ calories of SSB is higher than 543 calories.
